# Mental Health Professionals’ Views on Gaming to Inform Game-Based Interventions: Qualitative Cross-Sectional Study

**DOI:** 10.2196/69236

**Published:** 2026-04-20

**Authors:** Lauri Lukka, Veli-Matti Karhulahti, J Matias Palva

**Affiliations:** 1 Department of Neuroscience and Biomedical Engineering School of Science Aalto University Espoo Finland; 2 Department of Music, Art and Culture Studies University of Jyväskylä Jyväskylä Finland; 3 Neuroscience Center Helsinki Institute of Life Science University of Helsinki Helsinki Finland; 4 Centre for Cognitive Neuroimaging School of Psychology and Neuroscience University of Glasgow Glasgow United Kingdom

**Keywords:** clinical practice, digital interventions, mental health, mental health professionals, implementation science, qualitative study, serious games, thematic analysis, user-centered design, video games

## Abstract

**Background:**

Few game-based digital mental health interventions have been adopted in clinical practice, where mental health professionals (MHPs) play a critical role in the uptake of new technologies. Existing evidence suggests that MHPs’ views on game-based interventions and entertainment video games are mixed, reflecting broader tensions surrounding video games, which are perceived as both harmful and beneficial. However, the underlying reasons for these perceptions have remained unclear, even though they may substantially influence MHPs’ willingness to adopt or refuse new clinical solutions.

**Objective:**

This qualitative cross-sectional study investigates how MHPs view entertainment video games and gaming in clinical contexts. By examining these perceptions, the study aims to inform the implementation of game-based digital mental health interventions in health care.

**Methods:**

This study combined 3 qualitative interview datasets (n=19, n=16, and n=6) capturing Finnish MHPs’ views on video games and gaming, resulting in a combined sample of 41 participants, of whom 56% (n=23) were women and 59% (n=24) reported playing games. The interview data were analyzed using reflexive thematic analysis. Additionally, 2 post hoc analyses were conducted with complementary qualitative questionnaire data (n=80) collected alongside the first dataset.

**Results:**

In total, 3 themes were generated to reflect the MHPs’ views. First, personal recreation, clinically harmful: MHPs demonstrated a self-client attitude asymmetry, describing their own gaming primarily as positive and recreational, while associating clients’ gaming with problems. Second, adverse technology and meaningful culture: MHPs expressed attitudinal ambivalence, making sense of gaming through conflicting frames as both potentially harmful technology and meaningful culture. Third, holistic exploration of clients’ gaming: MHPs evaluated their clients’ gaming within the broader context of the clients’ lives. The first post hoc analysis reinforced the observed self-client attitude asymmetry, showing that MHPs associated their own gaming experiences with more positive and fewer negative meanings compared to their clients’ gaming. The second post hoc analysis suggested that MHPs expected game-based interventions to be approachable, motivating, and complementary to other treatments, and particularly suited for children and youth, people with strong digital competencies, and clients who were withdrawn.

**Conclusions:**

Existing qualitative research on MHPs’ perceptions of video games remains limited and has not examined clinicians’ broader sense-making of gaming to inform the implementation of game-based interventions. This study identifies how clinician education can build on MHPs’ existing perceptions while addressing potential misconceptions by clearly differentiating game-based interventions from entertainment games, clarifying their clinical aims and mechanisms of action, situating them within clients’ broader care and recovery processes, and challenging narrow assumptions about their target audience. Together, these contributions address a critical gap in the literature and inform strategies to improve clinician education, communication, and the adoption of game-based interventions in mental health care.

## Introduction

### Game-Based Interventions as a New Treatment Modality

Game-based digital mental health interventions use elements from entertainment games to achieve health-related aims [1]. They include both fully fledged serious games and gamified interventions [2], and here we focus on the former category, referred to as game-based interventions. The rationale for designing such interventions includes increasing intervention appeal to facilitate treatment access [3], increasing user engagement with digital interventions to alleviate the well-known challenge of low user engagement [4], and establishing new modes of treatment to complement psychological interventions and pharmacotherapy, which do not help all patients [5,6]. Preliminary evidence supports the efficacy of game-based interventions for depression [7], anxiety [8], and other mental disorders [9,10]. Game-based interventions include, for instance, SPARX for adolescent depression [11]; StarStarter for social anxiety disorder [12]; Embers the Dragon, a child-parent intervention supporting children’s emotional development [13]; and EndeavorRx, which has received Food and Drug Administration authorization for the treatment of pediatric attention-deficit/hyperactivity disorder [14]. However, most interventions are still in the research and development stage, few commercial game-based therapeutics are available, and few have been broadly or routinely integrated into clinical practice [15]. Best practices around their use are thus only emerging. The translation from the controlled research environments to real-world settings remains a central challenge for many digital interventions [16], including game-based interventions.

Beyond clinical efficacy, the success of game-based interventions depends on their acceptability [17-19]. The evaluation of acceptability has typically focused on end users [18], but in health care, clinical stakeholders also hold sway; they act as gatekeepers who recommend and prescribe digital interventions [20]. As health care providers’ attitudes and consequent behavior can make or break the implementation of novel digital tools [21,22], it is vital to understand their perceptions and consider them in intervention implementation [23-27]. This is particularly significant considering that game-based interventions suggest a change of meaning [28]—what looks like an entertainment video game can be therapeutic. Such a category shift offers opportunities for reconceptualizing and renewing mental health interventions [29], but it can also provoke hesitancy and resistance. Our aim in this study is to examine mental health professionals’ (MHPs’) views on entertainment video games and gaming in clinical contexts to inform the implementation of game-based interventions.

### MHPs’ Views on Games Are Mixed

Game-based interventions are still uncommon in clinical practice, and there are few studies examining MHPs’ views on them. Surveys indicate that only 12%-16% of psychotherapists know of the existence of such treatments, and only 1%-7% use them [30-32], while there are no data on the uptake of game-based interventions in the regional context of this study, Finland. Game-based interventions are perceived to offer opportunities for clients to engage with therapeutic content, foster self-efficacy and responsibility, and improve therapy access and patient motivation [31]. However, they are also viewed as potentially increasing avoidance and isolation, promoting addictive gambling habits, and devaluing therapy. Moreover, another study found that 5% of psychotherapists exhibit unfavorable and 10% highly favorable attitudes toward game-based interventions [32]. However, the previous studies do not capture the reasons for MHPs’ sentiments, which are particularly important to understand when implementing novel solutions in health care contexts.

MHPs’ mixed views on game-based interventions may arise from their perceptions of the underlying media, namely, video games. They may associate video games with addictive and violence-inciting potential, while simultaneously being open to using them in clinical contexts [33]. MHPs recognize both disadvantages and benefits related to gaming: “*Paradoxically, it seems that the way gaming benefits one individual may disrupt another and vice versa*,” summarize Alho and Mankinen [34]*.* Such conflicting attitudes mirror lay perspectives regarding video games [33,35] that they are associated with aggressive [36,37] and addictive behavior [38,39], while also being popular entertainment [40,41] and linked to positive effects on affective well-being [42], cognitive skills [43,44], and even intelligence [45,46]. Przybylski [47] encapsulates this attitudinal tension concisely: “*Whether electronic games are on balance a good or bad thing remains an open question, but is a topic about which people hold strong opinions*.” This contradiction may be a key distinction between MHPs’ views on game-based and non–game-based interventions and underscores the need for further qualitative research to explore the tensions MHPs may perceive. Because most Finnish MHPs do not have first-hand experience with game-based interventions, this study focuses on their views of entertainment video games, the medium on which these interventions are based, and the activity of playing entertainment video games, that is, gaming.

### Research Question

The research question guiding this study is as follows: How do MHPs view entertainment video games and gaming in clinical contexts? By examining these perceptions, the study aims to advance understanding of gaming in clinical contexts for researchers and designers involved in the creation, evaluation, and implementation of game-based digital mental health interventions in health care.

## Methods

### Research Design Overview

This qualitative cross-sectional study investigated the views of Finnish MHPs on entertainment video games and gaming. This aimed to inform the implementation of game-based interventions, where designers and developers interact with health care professionals and need to be mindful of their perceptions. Aligned with this aim, the research paradigm was interpretive [48], seeking to understand the meanings MHPs assign to video games and gaming. The study data included 3 distinct interview datasets, which were gathered independently by Lukka et al (dataset 1, n=19) [20], Alho and Mankinen (dataset 2, n=16) [49], and Siutila et al (dataset 3, n=6) [50], for a total of 41 MHP interviews. All 3 datasets concerned MHPs’ views on video games but used slightly different interview guides as described further in this study in detail. The combined datasets were analyzed using reflexive thematic analysis (RTA) [51,52], which led to the generation of 3 themes. After the interview data analysis, 2 post hoc analyses were conducted using questionnaire data (n=80) that were gathered with dataset 1.

### Ethical Considerations

The study and data gathering for dataset 1 were reviewed and approved by the Aalto University research ethics committee (D/508/03.04/2022), and the study was preregistered on the Open Science Framework [53]. The study involving the gathering of dataset 2 did not involve any of the 6 criteria that lead to ethics review in Finland, and therefore, ethics review was not required or applied for [54]. The study gathering dataset 3 was reviewed and approved by the Human Sciences and Ethics Committee of the University of Jyväskylä (80/13.00.04.00/2021).

Dataset 1 is stored securely on Aalto University servers with restricted access to only the researchers of this study, while datasets 2 and 3 are anonymized and stored securely in the Finnish Social Science Data Archive (FSD). The dataset 2 has been published in FSD with permission for secondary analysis [49], and the dataset reuse [55] was justified by the rationale that this study was guided by a different and more interpretive research question. The dataset 3 has also been published in FSD with permission for secondary analysis [50]. The dataset 3 reuse was justified because it was earlier analyzed via completely different research questions and analytical process, which focused primarily on players rather than MHPs.

In all 3 studies, the participants provided informed consent in Finnish. For studies generating datasets 2 and 3, the original informed consent allows for the secondary analysis without additional consent. No compensation was provided to the participants. All data have been reported in a manner that ensures no individual participant can be identified from the study results.

### Study Participants

The study participants were Finnish MHPs or psychology students (Table 1). Dataset 1 was gathered to study the role digital tools and video games had on Finnish MHPs’ clinical practices. Dataset 2 was gathered to study clinical psychologists’ and psychology students’ attitudes toward video games, problematic gaming, and gaming disorder [34]. The second author acted as the supervisor for this work. Dataset 3 was gathered to study the difference between 2 types of intensive play—one related to treatment-seeking and the other related to thriving well-being. The research effort was led by the second author (VMK) [56]. The primary participants in the study were people who played digital games actively and had sought help for their gaming-related problems, and those who actively played esports without health problems. These 2 samples of video game players were contextualized with interviews from Finnish MHPs (n=6) who had experience in treating at least 1 client with gaming-related health problems (3 MHPs were therapists, others were clinical psychologists).

**Table 1:** The demographic data of study participants within and across the three datasets.

Characteristic			Dataset 1		Dataset 2				Dataset 3		All datasets
			Interviewees 1-19 (MHPs^a^, n=19), n (%)		Interviewees 20-25 (psychology students, n=6), n (%)		Interviewees 26-35 (psychologists, n=10), n (%)		Interviewees 36-41 (MHPs, n=6), n (%)		Interviewees 1-41 (n=41), n (%)
**Gender**											
	Woman	13 (68)		3 (50)		6 (60)		1 (17)		23 (56)	
	Man	6 (32)		3 (50)		4 (40)		5 (83)		18 (44)	
**Age (y)**											
	18-29	1 (5)		0 (0)		4 (40)		0 (0)		5 (12)	
	30-39	3 (16)		0 (0)		4 (40)		0 (0)		7 (17)	
	40-49	6 (32)		0 (0)		1 (10)		0 (0)		7 (17)	
	50-59	7 (37)		0 (0)		1 (10)		0 (0)		8 (20)	
	60-66	2 (11)		0 (0)		0 (0)		0 (0)		2 (5)	
	Unknown	0 (0)		6 (100)		0 (0)		6 (100)		12 (29)	
**Years of working experience**											
	0	0 (0)		6 (100)		0 (0)		0 (0)		6 (15)	
	<5	4 (21)		0 (0)		7 (70)		0 (0)		11 (27)	
	5-10	3 (16)		0 (0)		1 (10)		0 (0)		4 (10)	
	10-15	2 (11)		0 (0)		1 (10)		0 (0)		3 (7)	
	>15	10 (53)		0 (0)		1 (10)		0 (0)		11 (27)	
	Unknown	0 (0)		0 (0)		0 (0)		6 (100)		6 (15)	
**Plays games**											
	Yes	9 (47)		3 (50)		6 (60)		6 (100)		24 (59)	
	No	10 (53)		3 (50)		4 (40)		0 (0)		17 (41)	

^a^MHP: mental health professional.

### Participant Recruitment

For dataset 1, the study participants were recruited using a questionnaire distributed through professional associations, health care organizations, social media, and snowballing. A total of 80 MHPs completed an online questionnaire; 46 (58%) wanted to participate only in the questionnaire, and 34 (43%) also in the interview. The first author (LL) contacted 24 MHPs in the latter group via email, primarily in registration order, and received 21 (88%) responses, of whom 2 withdrew before the interview. For dataset 2, the study recruitment occurred in 2 parts. First, psychology students were recruited through the university email list, which led to 8 responses, of whom 6 were interviewed. Then, gaming and nongaming licensed psychologists were recruited using social media and emails. For dataset 3, the participants were recruited through health care institutions and snowballing.

### Data Collection

For dataset 1, the first author (LL) conducted the semistructured interviews [57] with the initial aim of examining MHPs’ views and needs regarding game-based digital interventions. The researcher’s preinterview assumptions and perspectives are described in the study preregistration [53]. The original interview guide consisted of 4 sections—MHP background, views on digital interventions, views on digital games, and views and needs concerning game-based digital interventions. During the early interviews, it became evident that participants had very limited first-hand experience with digital interventions or game-based digital interventions. Consequently, the interview guide was revised (available in the study by Lukka et al [20]) to focus on MHP background, digital tools more broadly, and video games and gaming. The interviews were conducted as a dialogue in which meaning was actively cocreated, and shared understanding was confirmed through the interviews. Member checking was not conducted [58]. The interviews were conducted in Finnish via Zoom (Zoom Video Communications Inc) between May and September 2022, and they had an average duration of 57 (SD 9) minutes. They were audio-recorded with the interviewee’s explicit permission, and the first author (LL) transcribed the recordings verbatim for analysis.

For dataset 1, data sufficiency was evaluated using the concept of saturation, such that data collection was concluded when no substantively new information emerged. Saturation was evaluated through analytic memoing conducted during and after the interviews [59]. After 12 interviews, diminishing returns in new information were observed, and the first (LL) and second (VMK) authors agreed that 19 interviews were sufficient to address the original research questions. Data analysis then commenced, focusing first on the digital tools used by the MHPs, which were reported elsewhere [20]. This analysis did not examine MHPs’ views on video games and gaming, because this topic was found to be largely independent of how MHPs used digital tools in their clinical practice.

Subsequently, when preparing the analysis of MHPs’ perceptions of video games and gaming, the second author (VMK), who had been involved in the studies gathering datasets 2 and 3, proposed the use of data triangulation. Incorporating datasets 2 and 3 allowed for the inclusion of material gathered by different researchers and using slightly different interview guides and recruitment networks. Consequently, dataset 1 was complemented with these additional datasets to enable data source and investigator triangulation [60], thereby enhancing data richness and credibility.

For dataset 2, the development of the interview guide was guided by the aim of investigating psychology students’ and psychologists’ views on video games, problematic gaming, and gaming disorder. To study these topics, the interview guide (Alho et al [34]) included 4 sections, that is, background questions to explore participants’ clinical background and gaming experience; an adapted version of the Attitudes Toward Gambling Scale 8 [61], in which the word “gambling” was replaced by the word “gaming,” to study attitudes toward gaming; follow-up questions on attitudes toward video games; and questions about the new diagnostic category of gaming disorder. The interviews had an average duration of 35 minutes, and they were conducted in Finnish via Zoom between February and March 2021. The interviews were audio-recorded with permission from the interviewee and transcribed verbatim.

For dataset 3, the development of the interview guide was guided by the aim of contextualizing problematic and esports play through interviews with MHPs who had worked with at least 1 individual experiencing gaming-related problems. VMK and Miia Siutila conducted interviews with MHPs using a semistructured 12-item interview guide (Siutila et al [50]), which examined their work context, clinical experiences, practices related to treating clients who lived with gaming-related problems, their personal gaming experiences, and their perceptions of gaming disorders. The interviews had an average duration of 48 (SD 8) minutes, and they were conducted remotely via Zoom between June and November 2021. The interviews were recorded with the interviewees’ permission and transcribed verbatim.

### Interview Data Analysis

The interview data analysis approach was inductive, interpretive, and pragmatic. This approach was selected due to the characteristics of the research domain [62]; there is little previous research on MHPs’ attitudes toward video games and game-based digital interventions, and the existing findings are mixed [63]. Therefore, it was logical to proceed with a ground-up data analysis approach [64] while acknowledging that no research can be fully inductive [65]. The study paradigm was interpretive [48] and aimed to understand the meanings MHPs attributed to video games and gaming in their clinical contexts. The analysis was also influenced by the pragmatic aim of furthering game-based digital intervention development, which is derived from the pragmatist stance in health care [66], which aims to alleviate suffering and improve well-being.

The data was analyzed using RTA [51,52]. The methodological choice was based on our overall evaluation of the research aims, the quality and nature of the data, and our familiarity with the method. We chose to use RTA over coding reliability, thematic analysis, or codebook thematic analysis [65] due to our inductive and interpretive approach. RTA emphasizes the researchers’ active and reflexive role in the interpretation of data that occurs at the intersection of theoretical assumptions, analytic resources, and skills, and the data [65,67]. The researcher does not discover, but develops, constructs, and generates themes that are defined as “*patterns of shared meaning, united by a central concept or idea*” [65]. This should lead to a “*compelling, insightful, thoughtful, rich, complex, deep, and nuanced*” analysis, which acted as a quality criterion [68].

The first author (LL) conducted the analysis. It began with combining the datasets 1-3 and familiarization with them while making notes. Then, the combined data were coded using Atlas.Ti 23 software (ATLAS.ti GmbH). RTA emphasizes deep engagement with the data [65], and considerable time was given to generating the themes over a period of over half a year. During this time, the researcher engaged with the data, coded it, took a distance from it, and returned to review the themes several times. The second author (VMK) reviewed and helped revise the generated themes in 4 sense-making sessions with the first author (LL). Through this process, 3 themes were eventually considered to be meaningful and stable. They are illustrated with translated interview quotes and numbers (eg, #1) that connect them to a particular interviewee and dataset (Table 1).

We consider the combined sample and the data, diverse and adequate, to answer the research question. In RTA, it is not recommended to use the concept of saturation [69]. Given the exploratory nature of this study and the complexity of clinicians’ perceptions of the topic of gaming, this study does not capture all possible views on the topic. For instance, this study did not gather data on MHPs’ views on specific game platforms or genres, or on specific game-based interventions. However, we consider that the sample is sufficient to answer the research question of how MHPs view video games and gaming in clinical contexts. For reference, comparable qualitative research typically requires only 9-17 interviews [70,71], whereas our combined sample included 41 interviews. The inclusion of 3 complementary datasets enhanced the diversity and comprehensiveness of the qualitative material and enabled data source and investigator triangulation [60]. The manuscript was reviewed and revised in consultation with the Standards for Reporting Qualitative Research [72] (Multimedia Appendix 1) and Journal Article Reporting Standards for Qualitative Research [73].

The first author (LL) rooted the analysis in the data while being mindful that their own experiences in the domain inevitably contribute to their interpretations—in RTA, this awareness acts as a resource [65]. Considering positionality, the first author (LL) is a Finnish psychologist who has worked in Finnish mental health care, which allowed them to relate to the participant’s clinical working context. On the other hand, they are experienced in games; they play analog and digital games, have a degree in game design and production, and they have worked with entertainment games [74], gaming disorder [75], and game-based intervention for adult major depressive disorder [76]. This allowed them to connect with the participant’s personal and professional experiences regarding video games. The second author (VMK) is an interdisciplinary researcher of play and games, with a specific focus on clinical and psychological approaches to gaming. A personal history of active gaming has also contributed to the second author’s researcher position.

### Post Hoc Analysis

After the interview data analysis, 2 qualitative post hoc analyses were designed to provide complementary perspectives to the primary interview data analysis. The post hoc data were gathered with dataset 1 using an online questionnaire (Multimedia Appendix 2). The respondent characteristics were 80% (64/80) identified as women, 81% (65/80) were 30-59 years old, 74% (59/80) were psychologists, 78% (62/80) worked full time, 40% (32/80) worked in specialized health care, 39% (31/80) played video games daily or weekly, 35% (28/80) did not play video games at all, and the respondents had an average of 16 (SD 12) years of mental health working experience (Multimedia Appendix 3).

The first theme generated via RTA revealed how MHPs’ personal and professional gaming experiences differed, suggesting self-client attitude asymmetry. The gathered questionnaire data allowed for the methodological triangulation of this theme [60]. Therefore, the first post hoc analysis compared the questionnaire respondents’ attitudes toward video games in their personal lives (question 22: “*What does playing digital games mean to you?*”) and in their professional context (question 25: “*How does video game play exhibit itself in your client work?*”). All participants answered both questions. The analysis was conducted by deductively coding MHPs’ open-ended responses by their emotional valence from positive to neutral to negative [59]. Responses containing multiple positions were coded as including multiple categories. For example, the statement “*[Gaming is] a way to spend time, a way to fill time, fun activity, numbing, something that you can use to avoid things and that you can get addicted to*” was coded as both positive and negative. The results were reported as domain summaries [67]. Because these post hoc analyses were qualitative and nonstatistical and used to illustrate patterns of meaning, CIs for coding frequencies are not reported.

The 3 RTA themes did not provide insights into what kind of benefits game-based interventions could have and for whom. Therefore, we decided to complement the interview data analysis with the second post hoc analysis, which analyzed MHPs’ responses to the questions regarding the expected benefits of game-based interventions (question 27: “*What benefits in particular would you expect from game-based digital therapies?*”) and their target audience (question 30: “*For whom do you think game-based digital therapies would be most useful and why?*”). In total, 79 (99%) participants answered question 27, and all participants answered question 30. The open-ended responses were categorized with descriptive coding [59] and reported as domain summaries.

## Results

### Overview

The RTA of interview data generated 3 themes (Table 2).

**Table 2:** Reflexive thematic analysis of interview data generated 3 themes, which are illustrated with quotes.

Theme	Description	Illustrative quotes
Personal recreation, clinically harmful	MHPs’^a^ views on video games were influenced by the context of their gaming experiences. MHPs’ self-selected personal gaming experiences were typically positive and associated with recreation and pastime. In contrast, MHPs’ professional experiences with gaming occurred in a problem-focused clinical environment, which associated their clients’ gaming with mental health challenges, harm, and potential addiction. However, the client cases could also expand MHPs’ views on gaming.	“Games are close to my heart and I have played since I was a kid. My earliest memories are associated with gaming: I played Super Mario Bros 2 on the Nintendo Entertainment System when I was 4-5 years old.” [#14] “I have two children and they play video games. I see what they are doing, and I have learned about that world through them, and I know what it is about.” [#17] “In my work, I am mostly faced with the problems gaming causes.” [#1] “[In the clinical context] I have maybe seen the breadth of problematic gaming more than what I would have seen in a hobby. This has broadened my views on how different people benefit from and have problems with games.” [#26]
Adverse technology and meaningful culture	MHPs framed video games as both adverse technology and meaningful culture. Here, framing refers to a process of meaning-making through comparison and association. When MHPs framed video games as technology, gaming was associated with smartphones and digital media, which were perceived to have the potential for excessive use and harms. When MHPs framed video games as culture, gaming was compared to storytelling, hobbies, and arts, which were characterized as meaningful activities and recreation. Many MHPs acknowledged the simultaneous presence of both frames, which reflected the complexity of gaming as a societal phenomenon.	“We encourage people to read, watch movies, listen to music, and engage in other cultural experiences. I think games could very well be in the same category.” [#27] “I am occasionally frustrated that gaming is presented alongside snus, cannabis, and alcohol in a preventative substance abuse event. Gaming does not belong to that category, it is a hobby.” [#40] “Schools have pretty negative views on gaming. The teachers are concerned that children play a lot and spend a lot of time with digital devices.” [#32] “Games are not a black-and-white thing, but a new phenomenon to cope with. The same goes for smartphones, some people spend way too much time on them.” [#16]
Holistic exploration of clients’ gaming	MHPs explored their clients’ lives holistically: their responsibilities, social interactions, rest, exercise, and recreation. Particular attention was paid to whether any of these activities, including but not limited to gaming, had a detrimental effect on other life domains. Thus, the positive and adverse effects of any activity could only be identified by examining life as a whole. The balance between activities could change over time, and MHPs identified two courses where gaming could evolve from a recreational activity to a problem: from connection to loneliness and from comfort to avoidance.	“I try to think about life as a whole, about all the things that are important to the person. If you miss out on important because of playing, you play too much. This is true for other things as well: sleeping, working, seeing friends, and spending time with the spouse; so I don’t think it merely concerns playing.” [#33] “I think more important than the hours played is whether gaming has a negative influence on other fields of life.” [#24] “I don’t believe that [excessive] gaming is the root cause, but a symptom.” [#14] “Most people do not have problems [with gaming]. Those children who have, have other mental health problems or risk factors to them, such as anxiety disorders, social anxiety, maybe difficulties in forming friendships, domestic challenges.” [#29]

^a^MHP: mental health professional.

### Personal Recreation, Clinically Harmful

MHPs’ views on video games depended on whether they considered their own experiences (as players or spectators) or those of their clients. Gaming represented a long-term interest and hobby for several MHPs. One interviewee explained:

I have played quite a lot, for as long as I can remember. My first memory, in fact, is of a video game.

#23

Such a close relationship with gaming was understandably associated with perceiving the activity positively, even enthusiastically. Other MHPs played digital games more casually or had a more distant, spectator relationship with gaming. Their second-hand gaming experiences involved observing the gaming of their friends, siblings, spouse, and children, and such connections fostered familiarity with gaming and the perception of it as a meaningful recreational activity. Few MHPs associated video games with themes of threat and control, and they discussed the necessity of and challenges with limiting their children’s playtime to ensure moderation and daily rhythm.

The positive connotations associated with gaming in personal contexts were less present in problem-focused professional clinical contexts. Working with people who had mental health challenges often focused attention on problems, implicitly associating gaming with risks, harms, procrastination, interpersonal problems, and addictive behaviors. One participant reflected:

A psychotherapist always faces things that have become a problem. Then, the first association is that someone has a problem with games, that they are addicted or something.

#17

Such a perspective was reinforced by the clients having little need to explore gaming when it was recreational, positive, and meaningful. Therefore, MHPs’ professional perspective on gaming could be limited, and some felt their views were not representative of the general population. However, this did not necessarily mean their views were negative. Many MHPs acknowledged that gaming could offer their clients a meaningful way to pass the time, relax, socialize, and manage emotional states, but they commonly considered gaming a conflicted or divided topic that was shadowed by its potential harms.

MHPs’ personal gaming experiences were often self-affirmingly positive, whereas their professional context was associated with a greater possibility for attitudinal change and enrichment. Some MHPs, enthusiastic about video games, found that their professional experiences had made them more aware of potential problems associated with gaming. One participant described:

Before [running a video-game-related group intervention] I was more open-minded and liberal regarding problematic gaming. Now, I recognize and understand its divisiveness better: even if gaming is meaningful, it is potentially harmful.

#27

Conversely, a clinician with initial reservations about video games described how they came to appreciate the value gaming offered their clients:

If I was not working here at the neuropsychiatric clinic, I would probably think differently. I would think that games do more harm than good.

#9

These attitudinal changes stemmed from client cases that broadened MHPs’ perspectives beyond their usual social circumstances. Indeed, some MHPs reflected on case stories, which included cautionary examples, such as a compulsively playing mother and a boy walking into a ditch while playing on their phone, but also inspiring anecdotes, such as a physically disabled youth who found rich social contact through games and highly talented players who earned their living from competitive gaming. These exceptional case stories expanded MHPs’ views on gaming through idiosyncratic examples.

### Adverse Technology and Meaningful Culture

MHPs framed games as both adverse technology and meaningful culture. By framing, we refer to a process in which the interviewee associated and compared gaming with other activities, thus establishing categorical similarity and connection. This allowed MHPs to make sense of gaming by positioning it within broader societal developments and phenomena, which in turn also rationalized their attitudes and cyclically maintained them. Some MHPs emphasized one frame over another, while others acknowledged that gaming exhibited characteristics of both frames. This introduced tension and made it difficult to make sense of gaming as it was not merely harmful or beneficial, but rather had the simultaneous potential for both. Thus, gaming was a complex, divisive, and even polarizing topic that could not be described in black-and-white terms.

When MHPs framed video games as technology, they grouped and compared them with smartphones, television, streaming services, and social media. Video games were viewed as an inseparable part of societal technologization and digitalization, as well as the growing use of digital media and smartphones, in a negative sense. One interviewee reflected:

I think that the problems with video game playing are part of a broader discussion on the use of technology. – – The same types of addictive features are also present in other digital software, not only in games. Therefore, examining video games independently does not capture the whole they are a part of.

#23

Some MHPs considered this change to be linked to the technology and gaming industries, which aimed to design highly engaging products to increase their revenues while presenting themselves favorably as providers of employment and tax revenue. The frame of technology was associated with concerns about excessive digital device use, which could disrupt sleep, detach patients from their bodily experiences, and encourage social withdrawal. One participant explained:

I am afraid of what kind of a country or world this will become if there is too much digital interaction. It feels as if there is a technological religiosity, and digital playing is only a part of it.

#21

When gaming was framed as technology, it was associated with the disproportionate, pervasive, and adverse influence of digital devices on society.

In contrast, some MHPs framed video games as culture. They described gaming as a hobby, pastime, and recreational activity, which associated it with widely accepted activities such as storytelling, reading, doing crossword puzzles, listening to music, going to the movies, and doing handcrafts. This normalized and depathologized gaming and linked it with positive, life-enriching, and meaningful experiences, relaxation, and ways to pass the time. Video games were seen as the latest manifestation of the continuous cultural evolution of recreational activities. One interviewee described:

My generation played with dolls, cars, and in sandboxes—these activities have shifted [to video games].

#19

Concerns regarding the ill influence of gaming were perceived as an instance of media panic, where the latest form of media was unnecessarily feared and blamed. Within the frame of culture, the distinct feature of video games was their capability to invite participation in an immersive and sensory-rich story. One clinician characterized this as follows:

Video games are a form of art that enables processing different themes, telling stories, and stepping into another person’s shoes. They do it in a way that is different or unique compared to literature, movies, theater, or other forms of art.

#31

These features set video games apart from other forms of culture to which they were compared.

### Holistic Exploration of Clients’ Gaming

MHPs explored their clients’ lives holistically. They found that the universal pillars of a good life consisted of sufficient sleep, nutrition, exercise, social relationships, pursuing livelihood through studies or work, and recreation. MHPs consistently positioned video games within recreation where gaming offered challenges, feelings of competence, opportunities for social interaction, and a way to unwind and relax. However, they found that evaluating the overall impact of gaming required considering it against the other life domains, because well-being was a matter of balance. One participant described this as “*a golden middle way*” (#32). Another elaborated:

If you have a lot of social relationships, do sports, play an instrument, and then sometimes play video games, even a lot, things are in balance. [The influence of gaming] depends on the whole.

#21

Vice versa, MHPs agreed that disturbances to the balance between activities lead to problems. This meant that one activity, such as gaming, could cause “*harm to other fields of life*” (#9). This included negative influence on responsibilities, such as work or studies, or the fulfillment of basic human needs, such as nutrition, rest, daily rhythm, exercise, and social contact. Therefore, the activity itself was not perceived as a problem, but its influence on other necessary and intrinsically valuable domains of life. Moreover, the participants did not find that this principle was specific to video games, because anything in excess could be detrimental.

Therapeutic interaction included exploring the position and role video games played in the client’s life. The interviewees found that challenges in regulating gaming were rarely the primary reason for help-seeking, but other mental health issues, such as depression and anxiety, were. These symptoms, in turn, had predisposing factors, such as developmental and neuropsychological problems, learning disabilities, and external stressors (such as challenging domestic environments, bullying, bereavement, breakup, and loneliness). From this perspective, an existing behavioral problem, such as excessive gaming, could be a coping attempt to an emotional, social, or cognitive issue. Over time, the role of playing may have changed from an initial solution to a rigid, unsuccessful coping mechanism. One clinician characterized:

The motivation to playing may not be the same as it once was. It may be more compulsive and routinized, and you may not enjoy it as much.

#41

Thus, using games as self-help could turn from the alleviation of symptoms to the maintenance of the client’s issues.

MHPs described 2 problematic pathways in which initial recreational gaming could evolve into a problem—from connection to loneliness and from comfort to avoidance. Regarding the former, MHPs found that players may seek social connection from digital games, for instance, due to shyness or social anxiety. However, finding such online connections may lead to further withdrawal from social contacts outside gaming and exacerbate social anxiety, because the patients do not get exposure to real-life social situations. One clinician described:

Multiplayer games are an opportunity to find friends and community from the other side of the world – – but it can also offer an easy way out so that you don’t have to think about your feelings of loneliness and find other solutions as you can withdraw to the game world.

#31

Considering the latter pathway, the clients may also struggle with stress and depression, and fail to experience meaning in their lives, which can lead to seeking comfort, feelings of competence, and motivation from digital games. However, because gaming requires less effort than studying or working, this may lead to detrimental avoidance behavior, which prevents solving the underlying issues. Using gaming to manage stress, anxiety, and depression may end up aggravating the problems through self-defeating behavior. One clinician portrayed:

At least initially, gaming may have been therapeutic. You may have anxiety, depression, or a life crisis, and then you seek comfort from playing. But over time the amount of gaming may increase and become a problem of its own.

#37

Differentiating between gaming as an avoidance behavior and a coping strategy could be challenging, but it was considered important in understanding the influence of gaming.

### Post Hoc Analysis of Questionnaire Data

#### Do MHPs’ Views on Their Own and Their Clients’ Gaming Differ?

This first post hoc analysis triangulated the first theme generated in the RTA, investigating whether MHPs’ views on their own and their clients’ gaming differed. The post hoc analysis found that MHPs associated their own video game experiences with more positive meanings compared with their clients’ gaming (Table 3). When thinking about their own life, 73% (58/80) of MHPs associated gaming with positive meanings, such as recreation, relaxation, benefits, and a way to spend time, whereas considering their clients, only 38% (30/80) made such positive associations. Examining their own life, 16% (13/80) viewed video games in a negative light as uninteresting, uninspiring, addictive, and a waste of time, whereas when thinking about their clients, 64% (51/80) were concerned about excessive gaming, gaming-related interpersonal problems, avoidance behavior, and problems with daily rhythm. Overall, this analysis provided further evidence of the self-client attitude asymmetry concerning video games.

**Table 3:** The post hoc analysis of open-ended questionnaire responses (n=80) with quotes annotated with the respondent number (eg, #q1). Responses could be coded into multiple categories, and therefore percentages do not sum to 100.

Question and category			Quote		n (%)
“**What does playing digital games mean to you?” (question 22)**					
	Positive or beneficial	“A way to spend time with my spouse and friends. Pleasant recreation.” [#q54] “An opportunity to momentarily disrupt my thoughts by intensively focusing on something else.” [#q46]		58 (73)	
	Neutral	“I don’t have a relationship with playing.” [#q29] “It is not currently a part of my life at all. I last played in the 90s.” [#q17]		19 (24)	
	Negative or harmful	“They are something I don’t want to give any time from my life.” [#q6] “Senseless waste of time and a residue of childhood.” [#q64]		13 (16)	
“**How does video game play exhibit itself in your client work?” (question 25)**					
	Positive or beneficial	“Some clients consider playing a meaningful hobby.” [#q9] “It offers many people a way to relax like watching TV did earlier.” [#q39]		30 (38)	
	Neutral	“It only shows itself in conversations with clients.” [#q44] “Almost all clients play.” [#q21]		25 (31)	
	Negative or harmful	“People increasingly use these games to avoid real social contacts.” [#q6] “It maintains insomnia.” [#q20]		51 (64)	

#### What Benefits Could Game-Based Interventions Have and for Whom?

This second post hoc analysis complemented the RTA of interview data. It used the open-ended questionnaire responses to investigate what kind of benefits game-based interventions could have and for whom. Many MHPs (n=37, 46%) found that game-based interventions could be approachable and lower the threshold to participate in treatment. Some MHPs (n=20, 25%) expected that game-based interventions could create therapeutic impact by motivating the client to take care of themselves, be more active, build new skills, and self-reflect. Some MHPs (n=18, 23%) found that game-based interventions could augment and complement traditional therapies by providing structured content between sessions. Few MHPs (n=4, 5%) considered that game-based approaches were not feasible because they do not incorporate the crucial salutatory component of therapeutic, interpersonal interaction.

Many MHPs (n=34, 44%) considered that game-based interventions were particularly suitable for children and youth. This view was occasionally rationalized with younger generations being digitally native, and many MHPs (n=33, 41%) considered that competence with computers or playing video games could facilitate the uptake of game-based interventions. While the mentions of gender were infrequent, MHPs exclusively mentioned boys and men (n=6, 8%) when making such remarks. The participants also considered that game-based interventions could be feasible for clients who were hard to reach, withdraw, or have challenges with interpersonal interaction (n=25, 31%), or have neuropsychological challenges (n=5, 6%). In contrast to these cases that exhibited rather severe psychiatric problems, some participants suggested that the client’s symptoms should be mild and that the client should be proactive and capable of using such interventions (n=9, 11%). Refer to Multimedia Appendix 4 for quotes.

## Discussion

### Principal Findings

This qualitative study investigated Finnish MHPs’ views on gaming to inform the implementation of game-based interventions in health care. Although previous research highlights the importance of clinicians’ views on the adoption of new health care technologies [23-27], detailed accounts of how clinicians make sense of gaming within clinical contexts have remained limited. To address this gap, we analyzed 3 distinct qualitative interview datasets (n=41) using RTA, which converged into three themes: (1) MHPs exhibited a self-client attitude asymmetry where they associated their own gaming positively with recreation while linking their clients’ gaming to problems; (2) MHPs’ views expressed attitudinal ambivalence: they made sense of gaming through conflicting frames, viewing it simultaneously as harmful technology and meaningful culture; and (3) MHPs evaluated clients’ gaming holistically within the broader context of clients’ lives (Table 2). Furthermore, 2 complementary post hoc analyses of open-ended questionnaire responses (n=80) further contextualized these themes. The first corroborated the self-client attitude asymmetry identified in the interviews (Table 3). The second expanded the core findings by showing that MHPs expected game-based interventions to be approachable, motivating, and complementary to other treatments, and particularly suitable for children and youth, digitally native generations, and clients who were withdrawn or difficult to reach. Overall, these findings provide nuanced insights into the perceptions shaping MHPs’ decisions to adopt and recommend game-based interventions in clinical practice.

### Self-Client Attitude Asymmetry

In the first theme, we found that MHPs’ personal gaming experiences were positive and associated primarily with recreation. This aligns with previous work showing that limited gaming experience is associated with negative assumptions [47] and that first-hand exposure to gameplay can shift these views. Ferguson et al [77] demonstrated that older adults developed more positive attitudes toward video games after playing one themselves, concluding that “*negative attitudes toward video games exists mainly in the abstract and do not survive direct exposure to individual games*.” Because game-based mental health interventions are still rarely used in Finland, MHPs had no first-hand experience of such tools. Offering clinicians opportunities to try game-based interventions as they are introduced in health care may therefore help challenge preconceptions, improve acceptability, and support intervention uptake. These efforts may be further supported by lead users or internal champions [78] who have personal and professional experience of gaming and game-based interventions and can facilitate informed discussions within their organizations.

Previous research has shown that MHPs hold mixed views about gaming and game-based interventions [32,33], but the reasons underlying these perceptions have remained unclear. A key contribution of this study is the identification of a self-client attitude asymmetry; although MHPs viewed their own gaming positively, they tended to perceive their clients’ gaming as potentially problematic. This discrepancy may reflect the problem-oriented nature of psychiatric practice [79], where clinical encounters focus on difficulties and challenges, leaving the potential benefits of gaming underrecognized, even when they contribute to clients’ well-being. From an implementation perspective, these findings highlight the value of providing clinicians with concrete case examples that illustrate the therapeutic potential of game-based interventions.

### Negotiating Attitudinal Ambivalence

The second theme illustrates how MHPs use 2 nonexclusive and at times conflicting frames to make sense of gaming—adverse technology and meaningful culture. Entman [80] defines framing as selecting: “*some aspects of a perceived reality and make them more salient in a communicating text, in such a way as to promote a particular problem definition, causal interpretation, moral evaluation, and/or treatment recommendation*.” Thus, these frames allowed MHPs to interpret the complex phenomenon of video games within a broader societal context [81]. Previous research on serious games has identified 4 complementary frames [82]. This study expands the framing approach by focusing specifically on clinical contexts, revealing the 2 frames most salient to MHPs. Furthermore, these 2 frames exhibit attitudinal ambivalence [83], in which both positive and negative evaluations coexist, and suggest that MHPs’ perspectives on gaming cannot be accurately represented on a simple positive-negative continuum or measured with a single-dimensional scale.

The conflicting frames may surface in the implementation of game-based interventions, particularly when MHPs draw from broader societal discourses about the risks or cultural significance of video games and apply these discourses to game-based interventions. It may therefore be beneficial to acknowledge these frames and the complexity surrounding the issue of gaming, and to exhibit that the frames applied to entertainment gaming are not necessarily sufficient when considering game-based interventions; entertainment games are used for a range of recreational reasons [84,85], whereas game-based interventions are designed to achieve health benefits [1]. Clients’ expectations for game-based interventions are driven by a health-related need that the interventions aim to address through a therapeutic mechanism of action, which are not present in entertainment games. Thus, although game-based interventions may resemble commercial entertainment video games, they function as therapeutic tools or medical devices intended to meet specific health needs. Highlighting this distinction in clinician education and communications can help MHPs focus on the unique features of the technology and its intended purpose.

### Contextualizing Gaming

The third theme illustrates how MHPs adopt a holistic view when assessing clients’ gaming. They examined the role gaming played in clients’ lives and sought to differentiate problematic gaming from gaming that functions as a personal resource [86]. MHPs did not single out video games as a unique threat to well-being [63] but viewed gaming as one influence among many. When gaming presented problems, clinicians often explored how the meaning and motivations behind gaming had changed. Here, we identified 2 pathways through which recreational gaming could evolve into problematic gaming—from connection to loneliness and from comfort to avoidance. These pathways highlight that the functions of gaming are not static, but dynamic, shifting by the players’ life context.

The implementation of game-based interventions could benefit from adopting a similar holistic and temporal perspective. The educational and communication materials could emphasize the potential client-experienced benefits and position them within the broader context of their lives. To illustrate the role of game-based interventions in clients’ health care and recovery processes, implementation efforts could apply a patient journey approach [87]. Case examples may be particularly effective in demonstrating how game-based interventions match diverse life trajectories and contribute to the lived experience of recovery [88], thereby adding detail to health-related claims.

### Broadening Views on Users

The second post hoc analysis revealed that MHPs’ attitudes reflected common stereotypes about video game players as male, young, and socially withdrawn—perceptions that have been upheld by media representations [89]. References to player gender were exclusively about boys and men (Multimedia Appendix 4), consistent with the marginalization of female video game players. While men may more commonly identify as gamers [90], approximately half of video game players are female [41], and 59% of Finnish women play video games at least monthly [40]. Given that depressive and anxiety disorders are more common in women [91], it is likely that potential users of game-based interventions for mood disorders may be disproportionately female. This is consistent with our previous evaluation of a game-based intervention for adult depression, in which 57% (255/445) of participants identified as female, 34% (152/445) as male, and 7% (31/445) as nonbinary [76].

In this study, we also found common age-related stereotypes; many MHPs considered gaming to be an activity for children and youth. However, while the frequency of playing video games declines with age [40], an average player is 33 years old [41], and nearly half of Finns in their 30s play video games weekly [40]. Earlier, we found that a game-based intervention for adult depression attracted two-thirds of participants in their 20s and 30s, with a fourth aged 40-59 years [76], suggesting that there is an adult audience for these interventions.

Finally, some MHPs in this study associated video game playing with social anxiety, withdrawal, and awkwardness. However, previous work found no significant differences in social networks or exercise between online players, offline players, and nonplayers [89]. Overall, there appears to be a clear contrast between MHPs’ expectations and the actual characteristics of many game-based intervention users.

This finding has significant implications for the implementation of game-based interventions; their potential target audience may be broader than clinicians assume, and inaccurate stereotypes may inadvertently limit who MHPs recommend such interventions to. To expand understanding of actual intervention users, researchers may need to identify who engages with and benefits from game-based interventions. Target audience profiling [76] and user-journey analyses [92] can help broaden clinicians’ perceptions of the intervention user population and highlight cases in which interventions reach underrecognized patient groups, such as women older than 40 years.

### Limitations

This study was conducted in Finland, a digitally advanced country [93] where digital solutions are widely used, including in health care [94]. Finland also has an active entertainment game industry, employing 4100 people in 2022 [95], which may contribute to higher digital and gaming familiarity. Regarding representativeness, interview participation may reflect self-selection bias, as individuals with an interest in or favorable attitudes toward the topic may be more likely to participate [96]. The interviews did not capture MHPs’ perceptions of the regulatory status of digital interventions, which is a critical factor for their broader implementation. Regulatory frameworks differ across countries, are constantly evolving [97,98], and debate remains regarding which MHPs have the authority to prescribe such interventions [99], which warrant further investigation. Future research should also examine whether the identified frames and self-client attitude asymmetry are expressed in other cultural contexts. There are also opportunities for studying how MHPs’ perceptions may differ across certain gaming platforms and genres. Additionally, subsequent studies could broaden the analytical focus from individual MHPs to organizational factors, exploring how clinicians are introduced to and supported in using game-based interventions.

### Conclusions

Existing qualitative research on MHPs’ perceptions of video games is scarce and has not examined clinicians’ broader sense-making of gaming, limiting its usefulness for informing the implementation of game-based interventions. To fill this gap, this qualitative study explored the views of Finnish MHPs and suggests ways to improve clinician education by building on MHPs’ existing perceptions while addressing potential misconceptions. Because many MHPs have limited experience with game-based interventions, they tended to interpret them either through their own, often positive, recreational gaming experiences or through clients’ gaming-related problems, revealing a previously underexplored self-client attitude asymmetry. Their views also exhibited attitudinal ambivalence, such that they made sense of gaming through conflicting frames as both potentially harmful technology and meaningful culture. To alleviate these tensions, clinician education could more clearly differentiate game-based interventions from recreational entertainment games and highlight their clinical purpose, mechanisms of action, and expected benefits. This communication could be further enhanced by providing clinicians with first-hand exposure to these interventions and by illustrating their therapeutic potential with case examples.

MHPs also tended to evaluate the impact that video games had on their clients’ lives holistically and temporally, and they identified two pathways through which gaming could evolve into a problem: (1) from connection to loneliness and (2) from comfort to avoidance. Clinician education could use a similar approach and situate game-based interventions within clients’ broader health care and recovery processes. Finally, MHPs often assumed that game-based interventions are best suited for stereotypically male, young, and socially withdrawn individuals. Clinician communication could help challenge these preconceptions and demonstrate how game-based interventions are appealing and relevant to a wider range of users than MHPs may initially assume. Together, these findings address an important gap in the literature and offer practical guidance for improving clinician education, communication strategies, and the adoption of game-based interventions in mental health care.

## Data Availability

The dataset 1 used and/or analyzed during this study is available from the corresponding author on reasonable request. The dataset 2 supporting the conclusions of this article is available in the Finnish Social Science Data Archive repository (FSD3671). The dataset 3 supporting the conclusions of this article is available in the Finnish Social Science Data Archive repository (FSD3685).

## Appendix

Multimedia Appendix 1 Standards for Reporting Qualitative Research.

Multimedia Appendix 2 Questionnaire content.

Multimedia Appendix 3 Questionnaire respondents.

Multimedia Appendix 4 The second post hoc analysis summary - Game-based intervention benefits and target audience.

## Abbreviations

FSD: Finnish Social Science Data Archive
MHP: mental health professional
RTA: reflexive thematic analysis
