# Supplementary material for: Mental Health Professionals’ Views on Gaming to Inform Game-Based Interventions: Qualitative Cross-Sectional Study
Source: JMIR Serious Games. 2026 Apr 20;14:e69236. doi: 10.2196/69236 (PMC13139834; doi:10.2196/69236)
Supplement: Multimedia Appendix 1 [file games_v14i1e69236_app1.docx]

# Additional file 1 Standards for Reporting Qualitative Research

O’Brien, B. C., Harris, I. B., Beckman, T. J., Reed, D. A., & Cook, D. A. (2014). Standards for reporting qualitative research: a synthesis of recommendations. *Academic medicine*, *89*(9), 1245-1251.

| **No.** | **Topic** | **Description** |
| --- | --- | --- |
| **Title and abstract** | | |
| S1 | Title | “The title” describes the study topic and that it is qualitative.  “The abstract” describes that the study as qualitative; describes the data collection methods (qualitative interview and qualitative questionnaire data); and describes the qualitative data analysis method (reflexive thematic analysis). |
| S2 | Abstract | “The abstract” describes the key elements of the study: study background; objective; methods; results; and conclusions. |
| **Introduction** | | |
| S3 | Problem formulation | “The Introduction” includes three sections: (1) discussion of the problem and phenomenon studied; (2) existing prior research on the topic; (3) research question and study aim. |
| S4 | Purpose or research question | “Research questions” section at the end of “Introduction” includes the research question and study aims. |
| **Methods** | | |
| S5 | Qualitative approach and research paradigm | “Research design overview” section provides an overview to the qualitative approach and research paradigm. |
| S6 | Researcher characteristics and reflexivity | “Interview data analysis” section includes a paragraph on researcher characteristics and positionality. |
| S7 | Context | “Study participants,” and “Participant recruitment” sections include the description of the participants and data gathering contexts. |
| S8 | Sampling strategy | “Participant recruitment” section includes the description of sampling strategy. |
| S9 | Ethical issues pertaining to human subjects | “Ethical considerations” section includes the description of ethics board reviews. |
| S10 | Data collection methods | “Data collection” section describes the data collection and methods, including when and by whom the data collection was done. |
| S11 | Data collection instruments and technologies | “Data collection” section includes the instruments and technologies used in data collection, including the interview guides and devices. |
| S12 | Units of study | “Data collection” describes the number of participants and interview durations. |
| S13 | Data processing | “Data collection” describes how the data was processed, including the links to data storage when available. |
| S14 | Data analysis | “Interview data analysis” section includes description of how the themes were generated using reflexive thematic analysis.  “Post hoc analysis” describes how questionnaire data was analyzed. |
| S15 | Techniques to enhance trustworthiness | “Interview data analysis” section includes the description of used credibility strategies including data source triangulation, reflection, and sense-making sessions. |
| **Results / Findings** | | |
| S16 | Synthesis and interpretation | “Results” section reports the three generated themes. |
| S17 | Links to empirical data | “Results” section is substantiated with quotes that include references to Dataset 1–3. |
| **Discussion** | | |
| S18 | Integration with prior work, implications, transferability, and contribution(s) to the  field: | “Discussion” section examines the implication of the themes for game-based intervention implementation.  “The Discussion” section starts with a short summary of the main findings. |
| S19 | Limitations | “The Limitations” section includes reflection on the factors to consider when interpreting the study. |
| **Other** | | |
| S20 | Conflicts of interest | The “Conflicts of Interest” section describes this. |
| S21 | Funding | The “Funding” section describes this. |
