# Supplementary material for: Mental Health Professionals’ Views on Gaming to Inform Game-Based Interventions: Qualitative Cross-Sectional Study
Source: JMIR Serious Games. 2026 Apr 20;14:e69236. doi: 10.2196/69236 (PMC13139834; doi:10.2196/69236)
Supplement: Multimedia Appendix 2 [file games_v14i1e69236_app2.docx]

# Additional file 2

# Questionnaire content

# Questionnaire (translated)

**Research on the attitudes and needs of mental health professionals towards game-based digital therapies**

**Research information**

**Request to participate in the study**
We are asking you to participate in the research of the Aalto University Department of Neuroscience and Biomedical Engineering. This announcement describes what the study is about and what is required to participate in it. You can also ask the researcher for more specific details before the study or at any time during it.

**What are game-based digital therapies?**
Digital therapies are treatments based on clinical evidence, the aim of which is to diagnose, treat and promote the client's health. For example, the Mielenterveystalo.fi online therapy developed by HUS is a digital therapy.

Game-based digital therapies make use of elements from video games. These elements are, for example, points, goals, using stories and competitiveness. As far as we know, game-based digital therapies are not in use in Finland, but they are internationally.

You do not need to have experience with or using digital therapies or game-based digital therapies to participate in the study.

**What is the goal of the study?**
Game-based digital therapies are currently actively developed. Their development is based on previous research, where it has been found that playing video games can promote mental health and, among other things, alleviate the symptoms of depression. The goal of this research is to promote the development of new digital therapies in the form of games in the treatment of mental health. The study finds out what Finnish healthcare professionals think about using game-based digital therapies in mental health care and how these solutions could support the work of mental health professionals.

**Who can participate in the study?**
You can participate in the study if

1) you are a healthcare professional,

2) you work in mental health and

3) you have at least one client meeting per week.

**What does the research include?**
The research collects information in two parts: a questionnaire and a possible interview. You can participate only in the questionnaire survey or in both parts. Not everyone who is interested in an interview will necessarily be invited to an interview. Answering the questionnaire takes about 15 minutes. The interview is carried out remotely and lasts about an hour. The interview will be recorded for analysis. All research information is treated confidentially.

**What does research cost and who finances it?**
The measures related to the research are free of charge for the research subject and the research subject is not compensated. The research is financed by the Future Makers program of the Technology Industry Centenary Foundation and the Jane and Aatos Erko Foundation.

**What are the advantages and disadvantages of the research for the participant?**
The purpose of the study is to support the development of new digital therapies, and therefore it is not of direct benefit to the subject. There are no risks or disadvantages for the research subject except for the time spent on the research. Aalto University's Research Ethics Committee has pre-evaluated the research plan and approved it.

**Voluntary participation**
Participation in the study is voluntary. The subject can stop participating at any point before the end of the study without giving a reason or justification. Interrupting the study does not cause any harm to the subject. The subject can also withdraw his consent, in which case the data and samples collected from the subject will no longer be used in the analysis of new results from the moment of withdrawal. However, the materials that have already been processed remain as part of the material to ensure the research results.

**How does the research handle the data?**
The registrar of the study is Aalto University and the contact person of the registry is Lauri Lukka. The study's data protection notice can be read here: [link removed]

**Researchers' contact information**
If you have any questions about the study, you can contact the coordinating researcher or the study supervisor at any stage before, during or after the study's data collection. With them you can discuss all concerns you have during the research.

Coordinating researcher

Lauri Lukka

PhD researcher, psychologist

Department of Neuroscience and Biomedical Engineering,

Aalto University

+358 440 375 666,

[lauri.lukka@aalto.fi](mailto:lauri.lukka@aalto.fi)

Research supervisor

Matias Palva

Professor Department of Neuroscience and Biomedical Engineering,

Aalto University

+358 40 154 7779,

[matias.palva@aalto.fi](mailto:matias.palva@aalto.fi)

**Background information**

This questionnaire consists of four pages.

1. On this background information page, we go over, among other things, your education and work environment

2. The digital therapies section maps the use of digital therapies and your attitudes and expectations regarding them

3. The digital gaming section examines your gaming habits and your attitudes towards games

4. The game-based digital therapies section asks about your expectations and wishes regarding new forms of treatment

Answering the entire survey takes about 15 minutes. By answering that, you promote the development of new forms of treatment.

**1. Contact information**

First name:

Last name:

E-mail:

**2. Participation in research**

I only want to participate in the survey

I want to participate in both survey and interview research

**3. Your gender**

Woman

Man

Other

I do not want to say

**4 Your age**

18-29 years

30-39 years

40-49 years

50-59 years

60-69 years

70 years old

**5. Your background education (you can choose several)**

Specialized doctor

Practical nurse

Doctor

Psychologist

Psychotherapist

Nurse

Social worker

Sosionomi [A Finnish degree]

Other, what?

**6. How many years of work experience do you have in mental health work?**

**7. Which of the following best describes you?**

I work full time

I work part-time

I'm not working at the moment

**8. What is your current job title?**

[Open field]

**9. In which area do you primarily work?**

Etelä-Karjala

Etelä-Pohjanmaa

Etelä-Savo

Kainuu

Kanta-Häme

Keski-Pohjanmaa

Keski-Suomi

Kymenlaakso

Lappi

Pirkanmaa

Pohjanmaa

Pohjois-Karjala

Pohjois-Pohjanmaa

Pohjois-Savo

Päijät-Häme

Satakunta

Uusimaa

Varsinais-Suomi

**10. Where do you work?**

In primary health care

In special care

In occupational health care

In a rehabilitation facility

In school or student health care

As a private practitioner

In a research institute

Other, what?

**11. How many hours of direct client work do you have per week?**

[Number]

**12. Which of the following best describes your clients:**

They are healthy

They suffer from mild mental disorders

They suffer from moderate mental disorders

They suffer from severe mental disorders

**13. What are the most typical challenges your clients suffer from:**

[Open answer]

**Digital therapies**

Next, tell us a little about your thoughts regarding digital therapies. Digital therapies are treatments based on clinical evidence, the aim of which is to diagnose, treat and promote the client's health. For example, the Mielenterveystalo.fi online therapy developed by HUS is a digital therapy.

Digital therapies do not mean, for example, patient data registers that are not directly used in the treatment of patients.

**14. Does your workplace use digital therapies in the treatment of clients?**

Yes, what?

No

I do not know

I'm not in a work community

**15. Have you used digital therapies in your client work?**

Yes, what?

I don't

**16. How often do you use digital therapies in your work? ***

Daily

Weekly

About once a month

Rarely

Not at all

I can not say

**17. If you have used digital therapies in your client work, how have you and your client experienced using them?**

[Open answer]

**18. I find digital therapies useful ***

Completely agree

Somewhat agree

I can not say

Somewhat disagree

Completely disagree

**19. I think digital therapies are harmful ***

Completely agree

Somewhat agree

I can not say

Somewhat disagree

Completely disagree

**20. I wish digital therapies were used more ***

Completely agree

Somewhat agree

I can not say

Somewhat disagree

Completely disagree

**Digital gaming**

Next, tell us a little bit about your thoughts on digital gaming. In this context, digital gaming refers to **entertainment gaming** with, for example, a smartphone, computer or console. In this context, digital gaming does **not** mean money or gambling games.

**21. How often do you play digital games yourself? ***

Daily

Weekly

About once a month

Rarely

Not at all

I can not say

**22. What does playing digital games mean to you? ***

[Open answer]

Whether you play digital games or not, what do you think about the following statements?

**23. I think digital gaming is useful ***

Completely agree

Somewhat agree

I can not say

Somewhat disagree

Completely disagree

**24. I think digital gaming is harmful ***

Completely agree

Somewhat agree

I can not say

Somewhat disagree

Completely disagree

**25. How does digital gaming exhibit itself in your client work? ***

[Open answer]

**Digital therapies in the form of games**

The last section concerns game-based digital therapies in mental health care. There is currently very limited use of gamified digital therapies, so it is unlikely that you will have experience using them. This does not hinder answering the survey, because we are studying your attitudes and needs towards them.

In this section, game-based digital therapies are examined from four perspectives:
- What benefits do you think the therapies could have?- What mechanisms of action would you like the therapies to utilize?- For whom do you think the therapies could be most useful?- How could the therapies help your work?

**26. Game-based digital therapies are hypothesized to have several benefits.**

To what extent do you think the following statements are true? *

**Answer options**

- Completely agree
- Somewhat agree
- I can not say
- Somewhat disagree
- Completely disagree

**Questions:**

- **They lower the adoption threshold.** Game-based digital therapies are more attractive to adopt.
- **Encouraging the use of therapy.** Digital therapies in the form of games improve the fact that clients stay with the treatment and thus improve the effectiveness of the treatment.
- **New target groups.** Digital therapies in the form of games help to serve new and/or hard-to-reach target groups.
- **Reducing stigma.** Game-based digital therapies reduce the stigma associated with mental health disorders.
- **Remote care.** Digital therapies in the form of games enable remote therapy services.
- **Efficiency.** Digital therapies in the form of games ease the resource challenges of mental health services and improve the service system's ability to respond to customers' needs.

**27. What benefits in particular would you expect from game-based digital therapies?**

[Open answer]

**28. The effectiveness of game-based digital therapies can be based on many mechanisms.**

**Answer options:**

- Not impressive at all
- Not very impressive
- Somewhat impressive
- Impressive
- Very impressive

**Questions:**

- **Behavioral activation.** The form of treatment encourages the client to engage in meaningful activities.
- **Setting goals** . Therapy helps the client set goals for himself.
- **Self-understanding.** Therapy supports the development of the client's self-understanding and knowledge.
- **Cognitive training.** The therapy exercises the client's cognitive functions such as attentiveness and working memory.
- **Exposure therapy.** Therapy offers exposure exercises and therapy.
- **Cognitive behavioral therapy.** The therapy uses elements of cognitive behavioral therapy.
- **Awareness skills.** The therapy offers the client awareness skill exercises.
- **Biofeedback.** The therapy offers the client feedback on the functioning of his body.
- **Self-expression.** Therapy offers the client an opportunity for creativity and self-expression.
- **Peer support.** Therapy offers the client the opportunity to interact and connect with other clients.
- **Connection to a therapist.** Therapy offers a low-threshold communication channel with the therapist.

**29. If you wish, you can tell more about the answers above.**

What impact mechanisms would you like to use and why?

[Open answer]

**30. For whom do you think game-based digital therapies would be most useful and why? ***

[Open answer]

**31. Let's think about your work next.** How useful do you find the following features in game-based digital therapies:

**Answer options**

- Not helpful at all
- Not very helpful
- I can not say
- Useful
- Very useful

**The questions**

- **Psychoeducation.** I can share material related to mental health and well-being to my client.
- **Communication.** I can keep in touch with my client between meetings, for example by messaging.
- **Health monitoring** . I can monitor my client's symptoms, mood and well-being.
- **Effectiveness monitoring.** I can monitor the effectiveness of the treatment.
- **Psychological testing.** I can order, for example, neuropsychological, cognitive or personality mapping psychological tests via a digital platform.
- **Integration.** Game-style digital therapy integrates with existing patient information systems.

**32. How could game-based digital therapies most facilitate your work?**

[ Open answer ]

**33. What do you think about the use of game-based digital therapies in mental health care?**

I don't consider them plausible therapies at all - I consider them very plausible therapies

I don't consider them effective therapies at all - I consider them very effective therapies

**34. What else do you want to tell?**

[ Open answer ]

# Questionnaire (original in Finnish)

**Tutkimus mielenterveyden ammattilaisten asenteista ja tarpeista pelimuotoisia digitaalisia terapioita kohtaan**

**Tutkimuksen tiedote**

**Pyyntö osallistua tutkimukseen**
Pyydämme teitä osallistumaan Aalto-yliopiston neurotieteen ja lääketieteellisen tekniikan laitoksen tutkimukseen. Tässä tiedotteessa kuvataan, mistä tutkimuksessa on kyse ja mitä siihen osallistuminen edellyttää. Voitte myös kysyä tutkijalta tarkempia yksityiskohtia ennen tutkimusta tai koska tahansa sen aikana. 

**Mitä ovat pelimuotoiset digitaaliset terapiat?**
Digitaalisilla terapioilla tarkoitetaan tarkoitetaan kliiniseen näyttöön perustuvia hoitomuotoja, joiden tavoitteena on diagnosoida, hoitaa ja edistää asiakkaan terveyttä. Esimerkiksi HUSin kehittämä Mielenterveystalo.fi-nettiterapia on digitaalinen terapia.

Pelimuotoiset digitaaliset terapiat käyttävät hyväkseen digitaalisista peleistä tuttuja elementtejä. Niitä ovat esimerkiksi pisteet, tavoitteet, tarinoiden käyttäminen ja kilpailullisuus. Suomessa pelimuotoisia digitaalisia terapioita ei tietääksemme ole käytössä, mutta kansainvälisesti on. 

Sinulla ei tarvitse olla kokemusta digitaalisista terapioista tai pelimuotoisista digitaalisista terapioista tai niiden käytöstä osallistuaksesi tutkimukseen. 

**Mikä on tutkimuksen tavoite?**
Pelimuotoisia digitaalisia terapioita kehitetään tällä hetkellä aktiivisesti. Kehitys perustuu aikaisempaan tutkimustyöhön, jossa on havaittu, että digitaalisten pelien pelaaminen voi edistää mielenterveyttä ja muun muassa lievittää masennuksen oireita. Tämän tutkimuksen tavoitteena on edistää uusien pelimuotoisten digitaalisten terapioiden kehitystyötä mielenterveyden hoidossa. Tutkimus selvittää, mitä suomalaiset terveydenhuollon ammattilaiset ajattelevat pelimuotoisten digitaalisten terapioiden käyttämisestä mielenterveyden hoidossa ja miten nämä ratkaisut voisivat tukea mielenterveyden ammattilaisten työtä. 

**Kuka voi osallistua tutkimukseen?**
Voit osallistua tutkimukseen, mikäli 1) olet terveydenhuollon ammattihenkilö, 2) työskentelet mielenterveyden parissa ja 3) sinulla on vähintään yksi asiakastapaaminen viikossa. 

**Mitä tutkimus pitää sisällään?**
Tutkimuksessa kerätään tietoa kahdessa osassa: kyselylomakkeella ja mahdollisen haastattelun kautta. Voit osallistua vain kyselylomaketutkimukseen tai molempiin osuuksiin. Kaikkia haastattelusta kiinnostuneita ei välttämättä kutsuta haastatteluun. Kyselylomakkeeseen vastaaminen kestää noin 15 minuuttia. Haastattelu toteutetaan etänä ja se kestää noin tunnin. Haastattelu nauhoitetaan sen analysoimiseksi. Kaikkea tutkimustietoa käsitellään luottamuksellisesti. 

**Mitä tutkimus maksaa ja kuka sen rahoittaa?**
Tutkimukseen liittyvät toimenpiteet ovat tutkittavalle maksuttomia ja tutkimuksesta ei makseta tutkittavalle korvausta. Tutkimuksen rahoittaa Teknologiateollisuuden 100-vuotissäätiön ja Jane ja Aatos Erkon säätiön Future Makers -ohjelma. 

**Mitä hyötyjä ja haittoja tutkimuksesta on siihen osallistuvalle?**
Tutkimuksen tarkoitus on tukea uusien digitaalisten terapioiden kehitystä ja siten siitä ei ole suoraa hyötyä tutkittavalle. Tutkimuksesta ei ole tutkittavalle riskejä tai haittoja tutkimukseen käytettävää aikaa lukuun ottamatta. Aalto-yliopiston tutkimuseettinen toimikunta on ennakkoarvioinut tutkimussuunnitelman ja hyväksynyt sen. 

**Osallistumisen vapaaehtoisuus**
Tutkimukseen osallistuminen on vapaaehtoista. Tutkittava voi keskeyttää osallistumisensa missä tahansa vaiheessa ennen tutkimuksen päättymistä kertomatta syytä tai perustelua. Tutkimuksen keskeyttämisestä ei koidu tutkittavalle mitään haittaa. Tutkittava voi myös peruuttaa suostumuksensa, jolloin tutkittavasta kerättyjä tietoja ja näytteitä ei käytetä enää peruuttamishetkestä eteenpäin uusien tulosten analysoinnissa. Jo käsitellyt aineistot kuitenkin säilyvät osana aineistoa tutkimustulosten varmistamiseksi. 

**Miten tutkimuksessa käsitellään tietoja?**
Tutkimuksen rekisterinpitäjä on Aalto-yliopisto ja rekisterin yhteyshenkilö on Lauri Lukka. Tutkimuksen tietosuojailmoitus on luettavissa täällä: [link removed]

**Tutkijoiden yhteystiedot**
Jos teillä on kysyttävää tutkimuksesta, voitte olla yhteydessä koordinoivaan tutkijaan tai tutkimuksen ohjaajaan missä tahansa vaiheessa ennen tutkimuksen tiedonkeruuta, sen aikana tai sen jälkeen. Heidän kanssaan voitte keskustella kaikista tutkimuksen aikana mieltänne askarruttavista asioista.

Koordinoiva tutkija
Lauri Lukka
Väitöskirjatutkija, psykologi
Neurotieteen ja lääketieteellisen tekniikan laitos, Aalto-yliopisto
+358 440 375 666, lauri.lukka@aalto.fi

Tutkimuksen ohjaaja 
Matias Palva
Professori
Neurotieteen ja lääketieteellisen tekniikan laitos, Aalto-yliopisto
+358 40 154 7779, [matias.palva@aalto.fi](mailto:matias.palva@aalto.fi)

**Taustatiedot**

Tämä kysely koostuu neljästä sivusta. 

1. Tällä taustatiedot-sivulla käydään läpi muun muassa koulutustasi ja työympäristöäsi
2. Digitaaliset terapiat -osiossa kartoitetaan digitaalisten terapioiden käyttöä sekä asenteitasi ja odotuksiasi niitä koskien
3. Digitaalinen pelaaminen -osiossa tutkitaan pelaamistottumuksiasi ja asenteitasi pelejä kohtaan 
4. Pelimuotoiset digitaaliset terapiat -osiossa kysytään odotuksistasi ja toiveistasi uusia hoitomuotoja koskien

Koko kyselyyn vastaaminen kestää noin 15 minuuttia. Siihen vastaamalla edistät uusien hoitomuotojen kehittämistä.

**1. Yhteystiedot**

Etunimi:

Sukunimi:

Sähköposti:

**2. Tutkimukseen osallistuminen**

Haluan osallistua vain kyselytutkimukseen

Haluan osallistua sekä kysely- että haastattelututkimukseen

**3. Sukupuolesi**

Nainen

Mies

Muu

En halua sanoa

**4 Ikäsi**

18-29v

30-39v

40-49v

50-59v

60-69v

70v-

**5. Taustakoulutuksesi (voit valita useita)**

Erikoislääkäri

Lähihoitaja

Lääkäri

Psykologi

Psykoterapeutti

Sairaanhoitaja

Sosiaalityöntekijä

Sosionomi

Muu, mikä?

**6. Kuinka monta vuotta sinulla on työkokemusta mielenterveystyöstä?**

**7. Mikä seuraavista kuvaa sinua parhaiten?**

Olen töissä kokoaikaisesti

Olen töissä osa-aikaisesti

En tällä hetkellä työskentele

**8. Mikä on tämänhetkinen työnimikkeesi?**

**9. Millä alueella ensisijaisesti työskentelet?**

Etelä-Karjala

Etelä-Pohjanmaa

Etelä-Savo

Kainuu

Kanta-Häme

Keski-Pohjanmaa

Keski-Suomi

Kymenlaakso

Lappi

Pirkanmaa

Pohjanmaa

Pohjois-Karjala

Pohjois-Pohjanmaa

Pohjois-Savo

Päijät-Häme

Satakunta

Uusimaa

Varsinais-Suomi

**10. Missä olet töissä?**

Perusterveydenhuollossa

Erikoissairaanhoidossa

Työterveyshuollossa

Kuntoutuslaitoksessa

Koulu- tai opiskelijaterveydenhuollossa

Yksityisenä ammatinharjoittajana

Tutkimuslaitoksessa

Muu, mikä?

**11. Kuinka monta tuntia sinulla on suoraa asiakastyötä viikossa?**

[Numero]

**12. Mikä seuraavista kuvaa asiakkaitasi parhaiten:**

He ovat terveitä

He kärsivät lievistä mielenterveyden häiriöistä

He kärsivät keskivaikeista mielenterveyden häiriöistä

He kärsivät vaikeista mielenterveyden häiriöistä

**13. Mitkä ovat tyypillisimpiä haasteita, joista asiakkaasi kärsivät:**

[Avoin vastaus]

**Digitaaliset terapiat**

Kerro seuraavaksi hieman ajatuksistasi liittyen digitaalisiin terapioihin. Digitaalisilla terapioilla tarkoitetaan tarkoitetaan kliiniseen näyttöön perustuvia hoitomuotoja, joiden tavoitteena on

ddigitaalinen terapia. Digitaalisilla terapioilla ei tarkoiteta esimerkiksi potilastietorekisterejä, joita ei suoraan käytetä potilaiden hoidossa.

**14. Käytetäänkö työpaikallasi digitaalisia terapioita asiakkaiden hoidossa?**

Kyllä, mitä?

Ei

En tiedä

En ole työyhteisössä

**15. Oletko itse käyttänyt digitaalisia terapioita asiakastyössäsi?**

Kyllä, mitä?

En

**16. Kuinka usein käytät digitaalisia terapioita työssäsi? ***

Päivittäin

Viikottain

Noin kerran kuussa

Harvemmin

En lainkaan

En osaa sanoa

**17. Jos olet käyttänyt digitaalisia terapioita asiakastyössäsi, miten sinä ja asiakkaasi olette kokeneet niiden käyttämisen?**

[Avoin vastaus]

**18. Digitaaliset terapiat ovat mielestäni hyödyllisiä ***

Täysin samaa mieltä

Jokseenkin samaa mieltä

En osaa sanoa

Jokseenkin eri mieltä

Täysin eri mieltä

**19. Digitaaliset terapiat ovat mielestäni haitallisia ***

Täysin samaa mieltä

Jokseenkin samaa mieltä

En osaa sanoa

Jokseenkin eri mieltä

Täysin eri mieltä

**20. Toivoisin, että digitaalisia terapioita käytettäisiin enemmän ***

Täysin samaa mieltä

Jokseenkin samaa mieltä

En osaa sanoa

Jokseenkin eri mieltä

Täysin eri mieltä

**Digitaalinen pelaaminen**

Kerro seuraavaksi hieman ajatuksistasi digitaalisen pelaamisen osalta. Digitaalisella pelaamisella tarkoitetaan tässä yhteydessä **viihdepelaamista** esimerkiksi älypuhelimella, tietokoneella tai konsolilla. Digitaalinen pelaaminen ei tässä yhteydessä tarkoita raha- tai uhkapelejä.

**21. Kuinka usein itse pelaat digitaalisia pelejä? ***

Päivittäin

Viikottain

Noin kerran kuussa

Harvemmin

En lainkaan

En osaa sanoa

**22. Mitä digitaalisten pelien pelaaminen merkitsee sinulle? ***

[Avoin vastaus]

Riippumatta siitä, pelaatko digitaalisia pelejä tai et, mitä ajattelet seuraavista väitteistä?

**23. Digitaalinen pelaaminen on mielestäni hyödyllistä ***

Täysin samaa mieltä

Jokseenkin samaa mieltä

En osaa sanoa

Jokseenkin eri mieltä

Täysin eri mieltä

**24. Digitaalinen pelaaminen on mielestäni haitallista ***

Täysin samaa mieltä

Jokseenkin samaa mieltä

En osaa sanoa

Jokseenkin eri mieltä

Täysin eri mieltä

**25. Miten digitaalinen pelaaminen näkyy asiakastyössäsi? ***

[Avoin vastaus]

**Pelimuotoiset digitaaliset terapiat**

Viimeinen osio koskee pelimuotoisia digitaalisia terapioita mielenterveyden hoidossa. Tällä hetkellä pelimuotoisia digitaalisia terapioita käytetään hyvin rajallisesti, joten on epätodennäköistä, että sinulla on kokemusta niiden käytöstä. Tämä ei haittaa kyselyyn vastaamista, koska tutkimme asenteitasi ja tarpeitasi niitä kohtaan.

Tässä osiossa tarkastellaan pelimuotoisia digitaalisia terapioita neljästä näkökulmasta: 
- Mitä hyötyjä ajattelet, että terapioilla voisi olla?
- Mitä vaikutusmekanismeja toivoisit terapioiden hyödyntävän?
- Kenelle terapiat voisivat mielestäsi olla hyödyllisimpiä?
- Miten terapiat voisivat auttaa sinun työtäsi?

**26. Pelimuotoisilla digitaalisilla terapioilla oletetaan olevan useita hyötyjä. Missä määrin seuraavat väitteet pitävät mielestäsi paikkaansa? ***

**Vastaustavaihtoehdot**

- Täysin samaa mieltä
- Jokseenkin samaa mieltä
- En osaa sanoa
- Jokseenkin eri mieltä
- Täysin eri mieltä

**Kysymykset:**

- **Alentavat käyttöönottokynnystä.** Pelimuotoiset digitaaliset terapiat ovat houkuttelevampia ottaa käyttöön.
- **Terapian käyttöön innostaminen.** Pelimuotoiset digitaaliset terapiat parantavat sitä, että asiakkaat pysyvät hoidon parissa ja siten parantavat hoidon vaikuttavuutta.
- **Uudet kohderyhmät.** Pelimuotoiset digitaaliset terapiat auttavat palvelemaan uusia ja/tai vaikeasti tavoitettavia kohderyhmiä.
- **Stigman vähentäminen.** Pelimuotoiset digitaaliset terapiat vähentävät mielenterveyden häiriöihin liittyvää stigmaa.
- **Etähoito.** Pelimuotoiset digitaaliset terapiat mahdollistavat etämuotoiset terapiapalvelut.
- **Tehokkuus.** Pelimuotoiset digitaaliset terapiat helpottavat mielenterveyspalveluiden resurssihaasteita ja parantavat palvelujärjestelmän kykyä vastata asiakkaiden tarpeisiin.

**27. Mitä hyötyjä erityisesti toivoisit pelimuotoisilta digitaalisilta terapioilta?**

[Avoin vastaus]

**28. Pelimuotoisten digitaalisten terapioiden vaikuttavuus voi perustua moniin mekanismeihin.**

**Vastausvaihtoehdot:**

- **Ei lainkaan vaikuttavaa**
- **Ei kovinkaan vaikuttavaa**
- **Jossain määrin vaikuttavaa**
- **Vaikuttavaa**
- **Erittäin vaikuttavaa**

**Kysymykset:**

- **Käyttäytymisen aktivointi.** Hoitomuoto rohkaisee asiakasta mielekkääseen toimintaan.
- **Tavoitteiden asettaminen**. Terapia auttaa asiakasta asettamaan itselleen tavoitteita.
- **Itseymmärrys.** Terapia tukee asiakkaan itseymmärryksen ja -tuntemuksen kehittymistä.
- **Kognitiivinen harjoittelu.** Terapia harjoittaa asiakkaan kognitiivisia toimintoja kuten tarkkaavaisuutta ja työmuistia.
- **Altistushoito.** Terapia tarjoaa altistusharjoituksia ja -hoitoa.
- **Kognitiivinen käyttäytymisterapia.** Terapia käyttää kognitiivisen käyttäytymisterapian elementtejä.
- **Tietoisuustaidot.** Terapia tarjoaa asiakkaalle tietoisuustaitoharjoituksia.
- **Biopalaute.** Terapia tarjoaa asiakkaalle palautetta hänen kehonsa toiminnasta.
- **Itseilmaisu.** Terapia tarjoaa asiakkaalle mahdollisuuden luovuuteen ja itseilmaisuun.
- **Vertaistuki.** Terapia tarjoaa asiakkaalle mahdollisuuden vuorovaikutukseen ja yhteyteen muiden asiakkaiden kanssa.
- **Yhteys terapeuttiin.** Terapia tarjoaa matalan kynnyksen yhteydenpitokanavan terapeuttiin.

**29. Voit halutessasi kertoa lisää yllä olevista vastauksista. Mitä vaikutusmekanismeja toivoisit käytettävän ja miksi?**

[Avoin vastaus]

**30. Kenelle pelimuotoiset digitaaliset terapiat voisivat mielestäsi olla kaikkein hyödyllisimpiä ja miksi? ***

[Avoin vastaus]

**31. Ajatellaan seuraavaksi sinun työtäsi.** Kuinka hyödyllisinä pidät seuraavia ominaisuuksia pelimuotoisissa digitaalisissa terapioissa:

**Vastausvaihtoehdot**

- **Ei lainkaan hyödyllistä**
- **Ei kovinkaan hyödyllistä**
- **En osaa sanoa**
- **Hyödyllistä**
- **Erittäin hyödyllistä**

**Kysymykset**

- **Psykoedukaatio.** Voin jakaa asiakkaalleni materiaalia liittyen mielenterveyteen ja hyvinvointiin.
- **Yhteydenpito.** Voin pitää asiakkaaseeni yhteyttä tapaamisten välillä esimerkiksi viestitse.
- **Voinnin seuranta**. Voin seurata asiakkaani oireita, mielialaa ja vointia.
- **Vaikuttavuuden seuranta.** Voin seurata hoidon vaikuttavuutta.
- **Psykologinen testaus.** Voin teettää esimerkiksi neuropsykologisia, kognitiivisia tai persoonallisuutta kartoittavia psykologisia testejä digitaalisen alustan kautta.
- **Integroituminen.** Pelimuotoinen digitaalinen terapia integroituu olemassaoleviin potilastietojärjestelmiin.

**32. Miten pelimuotoiset digitaaliset terapiat voisivat eniten helpottaa sinun työtäsi?**

[ Avoin vastaus ]

**33. Mitä ajattelet pelimuotoisten digitaalisten terapioiden käytöstä mielenterveyden hoidossa?**

En pidä niitä lainkaan ukosttavina terapioina – Pidän niitä erittäin uskottavina terapioina

En pidä niitä lainkaan vaikuttavina terapioina – Pidän niitä erittäin vaikuttavina terapioina

**34. Mitä muuta haluat kertoa?**

[ Avoin vastaus ]
