# Supplementary material for: Mental Health Professionals’ Views on Gaming to Inform Game-Based Interventions: Qualitative Cross-Sectional Study
Source: JMIR Serious Games. 2026 Apr 20;14:e69236. doi: 10.2196/69236 (PMC13139834; doi:10.2196/69236)
Supplement: Multimedia Appendix 3 [file games_v14i1e69236_app3.docx]

# Additional file 3

# The demographics of questionnaire respondents (n = 80)

| **Variable** | **Category** | ***n*** | **%** |
| --- | --- | --- | --- |
| Gender | Woman | 64 | 80 |
|  | Man | 13 | 16 |
|  | Other | 1 | 1 |
|  | I don’t want to tell | 2 | 3 |
| Age | 18–29 | 5 | 6 |
|  | 30–39 | 21 | 26 |
|  | 40–49 | 24 | 30 |
|  | 50–59 | 20 | 25 |
|  | 60–69 | 9 | 11 |
|  | 70– | 1 | 1 |
| Education,  multiple may be chosen | Psychologist | 59 | 74 |
|  | Psychotherapist | 18 | 23 |
|  | Basic nurse or nurse | 17 | 21 |
|  | Doctor or specialized doctor | 3 | 4 |
|  | Other | 13 | 16 |
| Working status | Full time | 62 | 78 |
|  | Part-time | 17 | 21 |
|  | Not working | 1 | 1 |
| Working context | Specialized healthcare | 32 | 40 |
|  | Independent practitioner | 15 | 19 |
|  | School | 7 | 9 |
|  | Basic healthcare | 7 | 9 |
|  | Occupational healthcare | 6 | 8 |
|  | Other | 13 | 16 |
| Clients | Healthy | 2 | 3 |
|  | Mild mental disorders | 20 | 25 |
|  | Moderate mental disorders | 36 | 45 |
|  | Severe mental disorders | 22 | 28 |
| MHP frequency of video game playing | Daily | 12 | 15 |
|  | Weekly | 19 | 24 |
|  | Once a month | 6 | 8 |
|  | Less frequently | 15 | 19 |
|  | Not at all | 28 | 35 |
| Years of mental health work experience, mean (SD; range) |  | 15.6 y  (12.2,  1–45) |  |
| Hours of customer work per week, mean (SD, range) |  | 18.6 h  (7.3,  1–38) |  |
