# Supplementary material for: Mental Health Professionals’ Views on Gaming to Inform Game-Based Interventions: Qualitative Cross-Sectional Study
Source: JMIR Serious Games. 2026 Apr 20;14:e69236. doi: 10.2196/69236 (PMC13139834; doi:10.2196/69236)
Supplement: Multimedia Appendix 4 [file games_v14i1e69236_app4.docx]

# Additional file 4

# The second post hoc analysis summary:

# Game-based intervention benefits and target audience

| **What benefits in particular would you expect from game-based digital therapies? (question 27)** | | | |
| --- | --- | --- | --- |
| **Categories** | **Quotes** | ***n*** | **%** |
| Extending reach | *”Lowering the threshold for treatment, being easy to take up. Being interesting to children/youth. Efficiency, of course.”* (#q35).  *”I wish that game-based digital therapies could enable more people to access therapy. For instance, some marginalized youth play a lot of games, so gamifying therapy could be appealing to them.”* (#q56) | 37 | 46 |
| Creating therapeutic impact | *”It could encourage engaging in self-care and rest, doing daily chores, maintaining daily rhythm, going into nature, and being socially active.”* (#q18)  *”It could give experiences of success and participation, relaxation, delight, feelings of competence, because a depressed person does not feel much.”* (#q20) | 20 | 25 |
| Improving existing treatments | *”It could enable other psychosocial treatments to work.”* (#q16)  *”It could support the therapeutic process between and after the meetings.”* (#q38) | 18 | 23 |
| Don’t know | *“I don’t know.”* (#q28) | 5 | 6 |
| Resistance to game-based interventions | *”The lack of contact and meaning cannot be solved with a program that lacks interpersonal contact.”* (#q7)  *”I feel that game-based therapies lack central therapeutic elements, such as the possibility to engage in authentic interpersonal interaction with another person where you can process, for instance, your emotions and gain insight to your experiences.”* (#q68). | 4 | 5 |
| **For whom do you think game-based digital therapies would be most useful and why? (question 30)** | | | |
| **Categories** | **Quotes** | ***n*** | **%** |
| Children and youth | *”Children, youth”* (#q17)  *”Youth, because they are interested in game worlds.”* (#q23) | 34 | 43 |
| Digital native | *”People who are familiar with digital environments and who may already spend a lot of time there.”* (#q16).  *“Youth – they are naturally in the digital world and digitally native”* (#q39) | 33 | 41 |
| Withdrawing | *”People who are socially anxious, lonely, youth, afraid”* (#q10)  *”Clients who lack the skills to or dare not to verbalize their thoughts and feelings could benefit from support through a game world in the beginning of the treatment.”* (#47) | 25 | 31 |
| Mild symptoms | *”Clients who have mild mental health or addiction disorders.”* (#q26)  *”The problems should be mild (at maximum moderate). The client should have clear initiative, self-directedness, and motivation to process their challenges.”* (#q35). | 9 | 11 |
| Don’t know | *“I don’t know. Time will tell.”* (#q8)  *“I have so little knowledge on this that I don’t know.”* (#24) | 7 | 9 |
| Boys and men | *”Especially boys could benefit from them.”* (#q75)  *”Young relatively competent males, who would not otherwise be motivated.”* (#q49) | 6 | 8 |
| Neuropsychiatric challenges | *”Neuropsychiatric patients. Especially people with Asperger’s.”* (#q46)  *”Neuropsychiatric patients. Traditional approaches may not suite them, if they have difficulties in concentrating, self-reflection, or or abstract thinking.”* (#q72) | 5 | 6 |
